# Assessment of postoperative complications and their risk factors in general surgery using clavien-dindo classification

**DOI:** 10.6026/973206300212166

**Published:** 2025-07-31

**Authors:** Saumya Sinha, Deepak Pankaj, Mukesh Kumar, Rahul Kumar Sinha, Krishna Gopal

**Affiliations:** 1 Department of General Surgery, Indira Gandhi Institute of Medical Sciences, Patna, Bihar, India

**Keywords:** Post-operative complications, surgery, risk factors, incidence

## Abstract

Postoperative complications remain a significant challenge in general surgery, adversely impacting patient safety, hospital resources, and recovery outcomes. This study evaluated 800 patients using the Clavien-Dindo classification to assess the incidence and contributing risk factors. A complication rate of 31.5% was observed, with higher frequencies associated with comorbidities, increased BMI, emergency surgeries, prolonged operative time, infected wounds, and excessive intraoperative blood loss. The analysis identified statistically significant associations between these variables and complication severity. These findings underscore the importance of risk stratification, optimized surgical planning, and preoperative patient management to reduce postoperative morbidity.

## Background:

Unwanted outcomes of surgery, postoperative complications are a major source of concern that have a negative impact on patient safety and the standard of surgical care [1, 2- 3]. These range from ostensibly minor accidents that end without any harm to more significant ones that could endanger life, require numerous interventions, lengthen hospital stays and expenses and occasionally result in injury or death [4, 5-6]. Surgical complications can degrade a patient's quality of life and cause psychological stress in addition to physical harm. Complications must be reduced in order to raise the standard of surgical care [7, 8- 9]. A classification of surgical complications was proposed in 1992 by clavien *et al.* which was later modified in 2004 as clavien dindo classification [6, 8]. Initially its use in surgical practice was limited but in recent times its use is widely accepted from open surgical procedures to minimal invasive surgeries [7]. Therefore, it is necessary to comprehend the prevalence and risk factors of postoperative problems. Complications, however, are diverse and challenging to consistently capture [10, 11-12]. The sheer amount of surgical operations carried out each year is still increasing. With incidences of complications reaching as high around thirty percent in certain patient categories, many of these individuals will suffer from postoperative problems [13, 14- 15]. To enhance surgical results, efforts to enhance quality are growing in popularity. The ultimate objective is to reduce death and morbidity among patients by measuring results and pinpointing areas that require improvement [16, 17-18]. Enhancing quality healthcare favorable patient results are also of importance to payers as well as regulators. It is obvious that many of the things that patients care about the most will suffer as a result of postoperative problems [19, 20-21]. For instance, a patient's standard of life will probably suffer if they have pelvic sepsis after ileal pouch surgery. Although this kind of problem is severe and obviously would influence the standard of life, it is unclear whether other problems will have the same effect on outcomes that are patient-centered [22, 23]. Postoperative complications occur in about one-third of general surgery patients and are strongly associated with identifiable risk factors, highlighting the importance of patient optimization, surgical diligence, and infection control to improve outcomes and reduce complications [24]. As a result, we sought to ascertain if the research backs up the idea that complications following surgical procedures adversely affect other kinds of results that are patient-centered [25, 26, 27-28]. Therefore, it is of interest to describe the incidence and risk factors of postoperative complications in general surgery patients to guide the development of effective preventive strategies.

## Materials and Methods:

This study covered every patient who had been brought to the surgical department for either elective or urgent surgery after taking consent. Patients who had been transferred and underwent surgery somewhere else were not included. Intestinal endoscopies, childcare, and short-stay surgeries were excluded. This prospective study was conducted from October 2023 to October 2024 in Indira Gandhi institute of medical sciences, Patna. Information of the patient's clinical history, physical examination; laboratory reports, clinical diagnosis and surgery were recorded. Comorbidities, body mass index, gender and age were evaluated as risk factors for post-operative problems, surgical approach, surgery scheduling and American Society of Anesthesiologists grade as risk factors for postoperative problems. Hospital stay (days), class of wound, intraoperative complication, intraoperative blood loss (ml), duration of surgery (hours) were also included in the study. After surgery, patients were monitored for a period of thirty days, and any problems were recorded. Postoperative lengths of hospital stay were recorded. Any unwanted, unforeseen incident that directly results from an operation that affects the patient and would not have happened had the surgery gone as smoothly as could be reasonably anticipated was considered a complication. The Clavien-Dindo categorization was used to grade the problem (Table 1 - see PDF).

## Statistical analysis:

The experimental data was put in MS Excel. SPSS version 24 was used for statistical analysis. Chi square test and t test was used for statistical analysis. p value ≤ 0.05 was considered as statistically significant.

## Results:

In our study, 800 patients were evaluated and post-operative complications were reported in 252 patients. Frequency of post-operative complications was 31.5%. Among 252 patients most of the cases (146) were routine cases, (24) were urgent and (82) cases were of emergency nature. Maximum routine cases had grade 1 infection, followed by grade 2 with a few unfortunate cases having higher grade 4a and one routine case with grade 5. Most grade 4a complications followed by grade 4b complications were seen in emergency cases. And all except one grade 5 complications were of emergency laprotomy that landed in sepsis and died (Table 2 - see PDF). Comorbidities, Body Mass Index and age were found to significant variables for postoperative problems. Presence of comorbidities increased the possibility of postoperative problems (p=0.01). Increased BMI also increased the possibility of post-operative problems (p=0.001). 41-60 years patients were found to have maximum post-operative problems (Table 3 - see PDF). Surgical Approach, surgery Scheduling and American Society of Anesthesiologists grade were found to affect the possibility of post-operative problems. Open surgery had greater post-operative problems as compared to laparoscopic surgeries (p=0.048). Emergency surgeries were associated with greater post-operative problems (p=0.001). ASA grade III were related to greater post-operative problems (p=0.001) (Table 4 - see PDF). Hospital stay (days), class of wound, intraoperative complication, intraoperative blood loss (mL), duration of surgery (hours) were found to affect incidence of postoperative problems. Increased hospital stay increased the frequency of complications (p=0.001). Infected wounds were associated with greater incidence of post-operative problems (p<0.001). Presence of intra operative complications also increased the possibility of post-operative complications (0.02). Increased duration of surgery (p<0.001) and increased intraoperative blood loss (p<0.001) were related to increased frequency of post-operative complications (Table 5 - see PDF).

## Discussion:

The annual number of surgical procedures performed continues to rise. Many of these patients will experience postoperative issues, with some patient categories experiencing difficulties at rates as high as thirty percent [12, 13-14]. Efforts to improve quality are becoming more and more prevalent in an attempt to improve surgical outcomes. By tracking outcomes and identifying areas that need improvement, the ultimate goal is to lower patient mortality and morbidity [11, 12- 13]. Both payers and regulators place a high value on improving patient outcomes and the quality of healthcare [10, 11, 12, 13-14]. The objectives of this study were to determine incidence and risk factor general surgery department to obtain some clues to plan preventive strategies. In our study, 800 patients were evaluated and post-operative complications were reported in 252(31.5%) patients. Frequency of post-operative complications was 31.5%. Among 252 patients most of the cases 146 were routine cases followed by 24 urgent cases and 82 emergency cases. Based on clavien dindo classification, maximum routine cases had grade 1 infection, followed by grade 2 with a few unfortunate cases presenting with grade 4a complications. Most of the grade 4a complications were seen in emergency cases followed by grade4b complications. Only 1 routine case associated with comorbidities landed in death due to sudden cardiac arrest, hence designating it as grade 5. In our study, Comorbidities, Body Mass Index and age were found to significant variables for postoperative problems. Presence of comorbidities increased the possibility of postoperative problems (p=0.01). Increased BMI also increased the possibility of post-operative problems (p=0.001). 41-60 years patients were found to have maximum post-operative problems. The findings of our study are having similarity with the findings of other studies [25, 26, 27- 28]. These studies also reported that factors like presence of Comorbidities, increased Body Mass Index and age actively influence the incidence of post-operative problems [21, 22, 23-24]. The standard of surgical care and patient safety are negatively impacted by unintended surgical outcomes, or postoperative problems [20, 21, 22, 23- 24]. They range from seemingly insignificant mishaps that don't cause any harm to more serious ones that have the potential to endanger life, necessitate several procedures, prolong hospital stays and costs and even cause harm or death [24, 25-26]. In addition to causing bodily pain, surgical complications can lower a patient's quality of life and lead to psychological stress. To improve the quality of surgical care, complications must be decreased [17, 18- 19]. Understanding the frequency and risk factors of postoperative complications is therefore essential. However, complications are varied and difficult to reliably record [20, 21-22].

In our study, Surgical Approach, surgery Scheduling and American Society of Anaesthesiologists grade were found to affect the possibility of post-operative problems. Open surgery had greater post-operative problems as compared to laparoscopic surgeries (p=0.048). Emergency surgeries were associated with greater post-operative problems (p=0.001). AAA grade III was related to greater post-operative problems. The findings of our study are supported by findings of other studies [24, 25, 26-27]. It goes without saying that postoperative complications will negatively impact many of the things that patients value most. For example, pelvic sepsis following ileal pouch surgery is likely to negatively impact a patient's quality of life [21, 22-23]. Even while this type of issue is serious and would undoubtedly affect quality of life; it is uncertain if other issues will have the same impact on patient-centered outcomes [24, 25, 26- 27]. Therefore, we aimed to determine if the evidence supports the notion that post-operative complications negatively impact other patient-centered outcomes [13, 14, 15-16]. Hospital stay (days), class of wound, intraoperative complication, intraoperative blood loss (mL), duration of surgery (hours) were found to affect incidence of postoperative problems. Increased hospital stay increased the frequency of complications (p=0.001). Infected wounds were associated with greater incidence of post-operative problems (p<0.001). Presence of intra operative complications also increased the possibility of post-operative complications (0.02). Increased duration of surgery (p<0.001) and increased intraoperative blood loss (p<0.001) were related to increased frequency of post-operative complications. Comprehensive preoperative evaluation and therapy might lessen the detrimental effects of comorbidities. Physicians, anesthesia professionals and surgeons must coordinate and communicate well in order to manage comorbidities during surgery [23, 24, 25-26]. In literature, Bo lian et al established independent risk factor for overall complication based on clavien dindo classification [27]. It helped in predicting the clinical outcome based on severity [27, 28]. Although informative, it is seen that most of such studies are retrospective which forms a limitations of bais, especially recall bias that may occur. Hence prospective studies need to be done to get a better outlook.

## Conclusion:

Postoperative complications were observed in 31.5% of general surgery patients, with significant associations to modifiable and non-modifiable risk factors. Factors such as comorbidities, obesity, emergency procedures, and surgical complexity played a key role. Early identification and optimization of these risks can substantially improve surgical outcomes and patient safety.
